# Supplementary material for: End‐to‐End Crystal Structure Prediction from Powder X‐Ray Diffraction
Source: Adv Sci (Weinh). 2025 Jan 4;12(8):2410722. doi: 10.1002/advs.202410722 (PMC11848617; doi:10.1002/advs.202410722)
Supplement: Supplementary file 1 — Supporting Information [file ADVS-12-2410722-s001.pdf]

ADVANCED SCIENCE
Open Access

Supporting Information

for *Adv. Sci.*, DOI 10.1002/advs.202410722

End-to-End Crystal Structure Prediction from Powder X-Ray Diffraction

Qingsi Lai, Fanjie Xu, Lin Yao, Zhifeng Gao, Siyuan Liu, Hongshuai Wang, Shuqi Lu, Di He, Liwei Wang, Linfeng Zhang, Cheng Wang* and Guolin Ke**

Supplementary Information

Contents

A Supplementary Figures 2

B Supplementary Tables 3

A Supplementary Figures

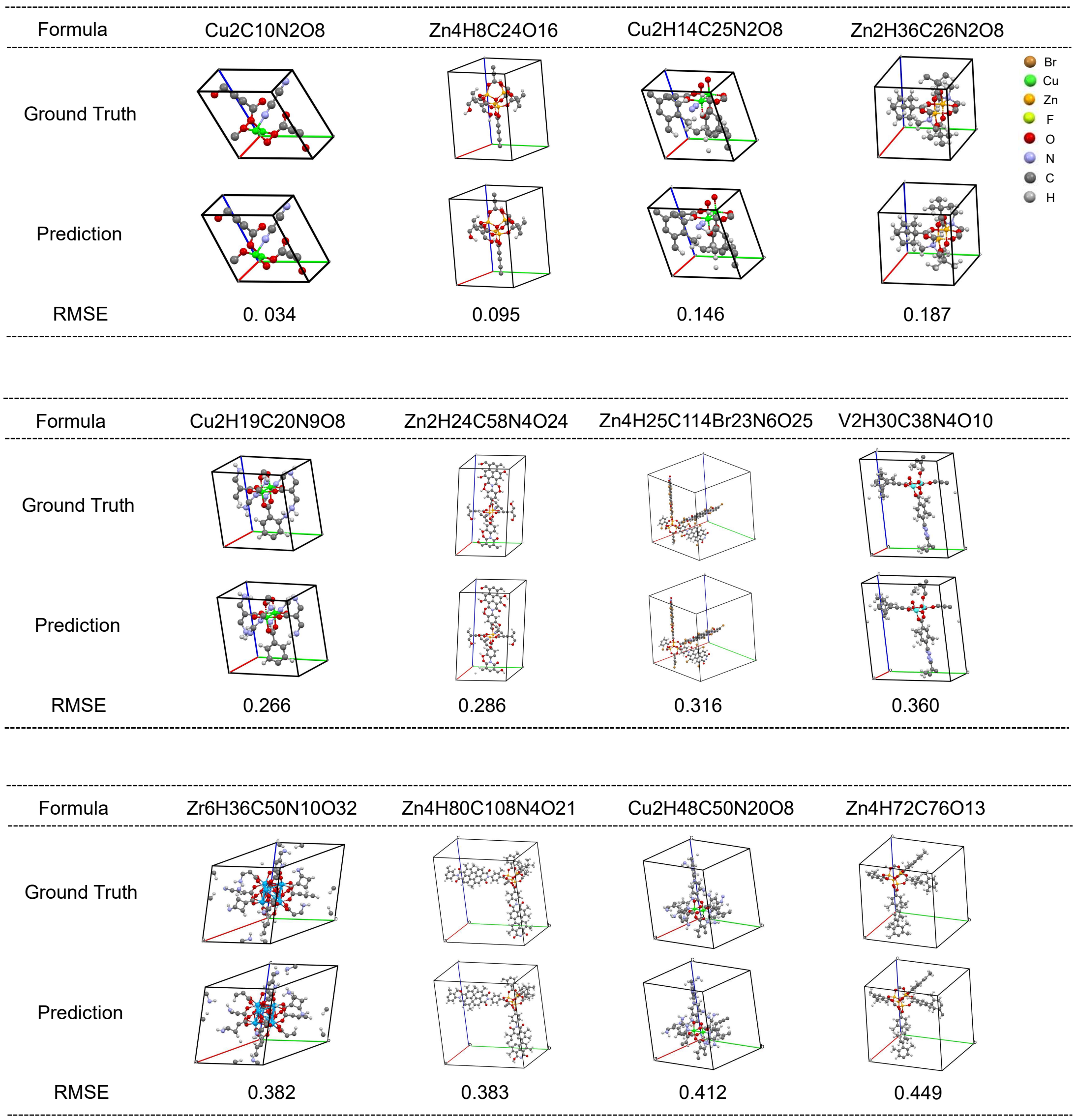

Supplementary Figure S1: More Visualizations of Prediction Structures and GT Structures.

B Supplementary Tables

Supplementary Table S1: Hit Rate for Database Retrieval

	Top-k			
	1	3	5	10
hMOF-100	46.0	78.1	89.4	97.2
hMOF-400	18.3	41.2	55.2	74.1

Supplementary Table S2: Match Rate and RMSE for Different Top Candidates.

Dataset	Metrics	Number of Candidates			
		1	3	5	10
hMOF-100	Match Rate ↑	64.3	80.7	85.7	90.2
	RMSE↓	0.424	0.401	0.387	0.366
hMOF-400	Match Rate ↑	42.7	65.5	72.0	79.0
	RMSE↓	0.481	0.458	0.446	0.430

Supplementary Table S3: Top-10 Match Rate and RMSE for Different Nerwork Design

Feat.	F	P	Match Rate ↑	RMSE↓
node			27.8	0.4749
cat			58.8	0.4032
cat		✓	88.5	0.3739
cat	✓	✓	90.5	0.378

Supplementary Table S4: Model Hyper-parameters for Crystal Structure Network

Hyper-parameters	
hidden dim	512
number of layers	6
time embed dim	256
time steps	1000
cosine scheduler s	0.008
σ_0, σ_T for $\boldsymbol{F}$	0.005, 0.5
step size in Langevin dynamics	1e-5

Supplementary Table S5: Model Hyper-parameters for PXRD Feature Extractor

Hyper-parameters	
encoder layers	8
embed dim	512
ffn dim	2048
attn heads	64
dropout	0.1
embed dropout	0.1
attn dropout	0.1
max seq length	2048
activation function	gelu
layer norm type	Pre-Norm

Supplementary Table S6: Optimization Hyper-parameters for CPCP Module Training

Hyper-parameters	hMOF-100	hMOF-400
optimizer	Adam	Adam
lr	5e-4	2e-4
β_1, β_2	0.9, 0.99	0.9, 0.99
eps	1e-6	1e-6
weight decay	1e-4	1e-4
lr scheduler	CosineWarmupScheduler	CosineWarmupScheduler
total epoch	400	400
warmup epoch	20	20
batch size	64	32
gradient clip form	norm	norm
gradient clip value	5	5

Supplementary Table S7: Optimization Hyper-parameters for CCSG Module Training

Hyper-parameters	hMOF-100	hMOF-400
optimizer	Adam	Adam
lr	1e-3	1e-3
β_1, β_2	0.9, 0.999	0.9, 0.999
eps	1e-8	1e-8
weight decay	0	0
lr scheduler	CosineWarmupScheduler	CosineWarmupScheduler
total epoch	400	400
warmup epoch	20	20
batch size	64	16
gradient clip form	norm	norm
gradient clip value	5	5
